# A Solid-State Wire-Shaped Supercapacitor Based on Nylon/Ag/Polypyrrole and Nylon/Ag/MnO_2_ Electrodes

**DOI:** 10.3390/polym15071627

**Published:** 2023-03-24

**Authors:** Ruirong Zhang, Xiangao Wang, Sheng Cai, Kai Tao, Yanmeng Xu

**Affiliations:** 1 Ministry of Education Key Laboratory of Micro/Nano Systems for Aerospace, School of Mechanical Engineering, Northwestern Polytechnical University, Xi’an 710072, China; 2 Cleaner Electronics Group, College of Engineering, Design and Physical Sciences, Brunel University London, Uxbridge UB8 3PH, UK

**Keywords:** wire-shaped asymmetric supercapacitor, polypyrrole, MnO_2_, nylon, wearable electronics

## Abstract

In this work, a novel wire-shaped supercapacitor based on nylon yarn with a high specific capacitance and energy density was developed by designing an asymmetric configuration and integrating pseudocapacitive materials for both electrodes. The nylon/Ag/MnO_2_ yarn was prepared as a positive electrode by electrochemically depositing MnO_2_ on a silver-paste-coated nylon yarn. Additionally, PPy was prepared on nylon/Ag yarn by chemical polymerization firstly to enlarge the surface roughness of nylon/Ag, and then the PPy could be easily coated on the chemically polymerized nylon/Ag/PPy by electrochemical polymerization to obtain a nylon/Ag/PPy yarn-shaped negative electrode. The wire-shaped asymmetric supercapacitor (WASC) was fabricated by assembling the nylon/Ag/MnO_2_ electrode, nylon/Ag/PPy electrode and PAANa/Na_2_SO_4_ gel electrolyte. This WASC showed a wide potential window of 1.6 V and a high energy density varying from 13.9 to 4.2 μWh cm^−2^ with the corresponding power density changing from 290 to 2902 μW cm^−2^. Meanwhile, because of the high flexibility of the nylon substrate and superior adhesion of active materials, the WASC showed a good electrochemical performance stability under different bending conditions, suggesting its good flexibility. The promising performance of this novel WASC is of great potential for wearable/portable devices in the future.

## 1. Introduction

With the prosperous development of wearable/portable electronics, energy storage devices are also being developed to be smaller, lighter and more flexible simultaneously. Flexible supercapacitors have attracted more attention because of their fast charge/discharge rate, high power density, long life cycle and good flexibility [1,2]. Among the different types of flexible supercapacitors, wire-shaped flexible supercapacitors have advantages such as small dimension, light weight, and they can be easily integrated with fabrics; therefore, they are considered to be the ideal energy storage devices for wearable/portable devices [3,4].

Among the components of wire-shaped flexible supercapacitors, the selected substrate greatly affects the overall performance of the device, such as the weight, flexibility and stretchability. In previous studies, metal wires and carbonaceous fibers have been extensively used in flexible supercapacitors because of their brilliant electrical conductivity and flexibility [5,6,7,8]. However, the metal wires are usually heavy, and the carbonaceous fibers have poor stretchability and are expensive; these characteristics have limited their applications in wearable/portable devices [9]. Compared with metal wires and carbonaceous fibers, commercial yarns, such as nylon yarn, are considered to be appropriate substrates for flexible wire-shaped supercapacitors because of their excellent flexibility, high tensile strength and low cost [10]. However, it is a challenge to change nylon yarns from insulators to electrical conductors [10]. Choi et al. [11] prepared CNT/nylon by wrapping carbon nanotube aerogel sheet ribbons on the surface of nylon yarns and changed the nylon yarns from insulators to electrical conductors, and then MnO_2_ was electrochemically deposited on the CNT/nylon yarn to obtain a MnO_2_/CNT/nylon fiber electrode, which showed an areal specific capacitance of 40.9 mF cm^−2^. Because of the symmetric structure, this supercapacitor showed a relatively narrow operating voltage window of 0–1.0 V, eventually resulting in a limited energy density. In fact, low energy density has always been a common weakness of flexible wire-shaped supercapacitors, which largely limits their further applications.

According to the calculation formula of the energy density (E = 1/2CU^2^), one of the effective methods to improve the energy density of supercapacitors is to expand their operating voltage window [12], which can be achieved by applying active materials with different operating voltage ranges on positive and negative electrodes separately to construct asymmetric supercapacitors [13]. For instance, Xu et al. [14] assembled a wire-shaped supercapacitor with an MnO_2_/CNT-positive electrode and aerogel CNT fiber-negative electrode together; the potential window of the device was extended from 0.8 to 1.5 V and the energy density was tripled from 11.47 to 39.85 nWh cm^−1^. The other typical method to improve the energy density of a device is to enlarge its specific capacitance. Pseudocapacitive materials normally involve metal oxides (such as MnO_2_ [15], Fe_2_O_3_ [16] and TiO_2_ [17]) and conducting polymers for supercapacitors (such as polyaniline [18,19,20,21] and polypyrrole [22]), which usually provide much higher specific capacitance than those of carbon-based materials used in electrical double-layer capacitors (EDLCs) [21]. Adopting active materials with pseudocapacitive properties on both positive and negative electrodes is an effective way to increase the capacitance of asymmetric supercapacitors [23]. Yuan et al. [24] fabricated a fiber-shaped asymmetric supercapacitor by using pseudocapacitive active materials CNT@NiO@MnO_x_ as cathodes and CNT@Fe_2_O_3_ as the anode. The potential window of this supercapacitor successfully extended to 1.8 V in an aqueous electrolyte, and it showed a high specific capacitance of 29.3 mF cm^−2^ and an energy density of 13.2 μWh cm^−2^, which can be greatly attributed to the brilliant pseudocapacitive performance of active materials in both positive and negative electrodes.

Herein, a wire-shaped asymmetric supercapacitor was created by placing two nylon-based electrodes in parallel. The positive electrode was prepared by electrodepositing MnO_2_ on a nylon/Ag yarn. Meanwhile, PPy was successfully grown on the nylon/Ag yarn by two steps, i.e., chemical polymerization and electrochemical polymerization, to obtain the negative electrode. Both electrodes with pseudocapacitive active materials had large operating potential windows (0–0.8 V for the nylon/Ag/MnO_2_ electrode and −0.8–0 V for the nylon/Ag/PPy electrode), which are beneficial to widen the working potential range of the assembled supercapacitor, e.g., from 0 to 1.6 V. The wire-shaped asymmetric supercapacitor showed a high linear specific capacitance (C_L_) of 38.9 mF cm^−1^ and an areal specific capacitance (C_A_) of 181.7 mF cm^−2^ at the scanning rate of 5 mV s^−1^, along with a six-fold energy density increase due to the expansion of the potential window from 0.8 V to 1.6 V. The fabricated supercapacitor also showed good flexibility and electrochemical stability under the bending state. Furthermore, the fabricated supercapacitors were connected and tested in series and in parallel to increase the operating voltage and current to meet the practical demands of different applications.

## 2. Materials and Methods

### 2.1. Materials and Reagents

Commercially available nylon yarn with a tensile stress of 283.8 MPa (Figure S1) was purchased from Chunguang Group Co. (Linyi, China). Conductive silver paste (GY-Ag-6811, 20,000–28,000 cp) was purchased from Sino-Platinum Metal Co., Ltd. (Guizhou, China). Manganese (II) acetate tetrahydrate (Mn(CH_3_COO)_2_·4H_2_O, 98%), pyrrole monomer (Py, 99%), ferric chloride (FeCl_3_, 98%) and sodium polyacrylate (PAANa, Mw: 30,000,000) from Adamas Co. (Shanghai, China) were selected. Sodium sulfate (Na_2_SO_4_, 99%) and ethanol (75%) were supplied by Greagent Co. (Shanghai, China). All chemicals were used without further purification. Deionized water (DI water) was used for all experiments and cleaning steps.

### 2.2. Preparation of Nylon/Ag/MnO_2_ Yarn Electrode

**Figure 1:**
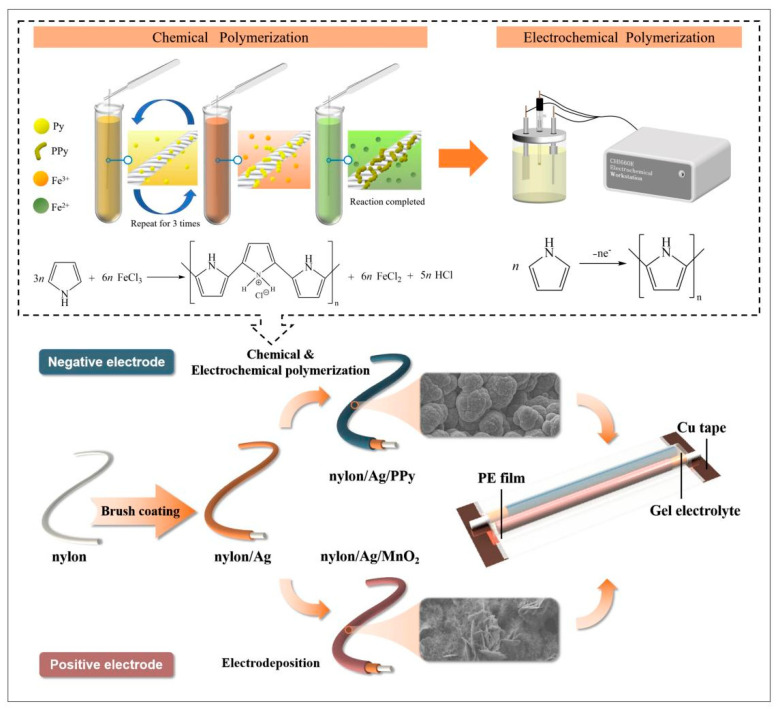
Schematic diagram of the fabrication process of nylon/Ag/MnO_2_ and nylon/Ag/PPy electrodes and the assembly of the asymmetric supercapacitor based on these two electrodes (the inset in the upper part shows the mechanism of PPy polymerization and the fabrication of the nylon/Ag/PPy electrode).

The fabrication processes of the nylon/Ag/MnO_2_ and nylon/Ag/PPy electrodes are illustrated in Figure 1. The nylon/Ag/MnO_2_ fiber electrode was fabricated by the electrochemical deposition (ECD) method on the nylon/Ag yarn. Firstly, the commercial nylon yarn (6 cm) was washed by DI water and immersed in ethanol for 15 min. Secondly, after the nylon yarn was naturally air dried, the conductive silver paste was uniformly coated onto the nylon yarn via the brush coating method. The silver-covered yarn was then put into a hot oven (DHG-9011A, from JingHong, Shanghai, China) at 80 °C for 1 h to cure the silver paste. When the yarn was cooled to room temperature, the second step was repeated once more to obtain the nylon/Ag yarn. The total mass of silver slurry coated on the nylon yarn was about 14 mg. After heat curing, the silver paste showed an excellent linear resistance of about 0.8 Ω cm^−1^. The optical pictures of the nylon/Ag yarn before and after the heat curing are exhibited in Figure S2a. The electrochemical deposition was carried out in a three-electrode electrochemical system (CHI660E electrochemical workstation, Chenhua, Shanghai, China) with the nylon/Ag as the working electrode, a platinum foil as the counter electrode and a saturated calomel electrode (SCE) as the reference electrode. A mixed aqueous solution of Na_2_SO_4_ (0.1 M) and Mn(CH_3_COO)_2_·4H_2_O (0.1 M) was used as the electrolyte. A constant potential of 0.8 V was applied to the working electrode for 1800 s. Then, the electrochemically deposited nylon/Ag/MnO_2_ electrode (600 μm in diameter, 0.94 cm^2^ effective surface area) was fabricated and flushed by DI water repeatedly and dried at room temperature.

### 2.3. Preparation of Nylon/Ag/PPy Fiber Electrode

The nylon/Ag/PPy yarn electrode was also fabricated based on nylon/Ag that was prepared as described in Section 2.2. Firstly, the nylon/Ag yarn was immersed in a mixed solution of 0.2 M NaClO_4_ and 6% (*v*/*v*) Py for 30 s and then soaked in a 0.3 M FeCl_3_ solution for 2 min. After that, the yarn was flushed with DI water to remove excessive FeCl_3_ solution. This process was repeated 3 times to obtain the nylon/Ag/PPy yarn electrode. Then, the electrochemical polymerization was carried out in the three-electrode electrochemical system with the chemically polymerized nylon/Ag/PPy electrode as the working electrode, a platinum foil as the counter electrode and a SCE as the reference electrode. The electrochemical solution contained 0.2 M NaClO_4_ and 5% (*v*/*v*) Py. A constant voltage of 0.8 V was applied for 400 s during the electrodeposition process. The obtained nylon/Ag/PPy electrode (765.7 µm in diameter, 1.20 cm^2^ effective nylon/Ag/PPy surface area) was flushed by DI water repeatedly after polymerization and then dried at room temperature.

### 2.4. Fabrication of Solid-State Asymmetric Supercapacitor

The nylon/Ag/MnO_2_||nylon/Ag/PPy solid-state wire-shaped asymmetric supercapacitor was fabricated as follows (Figure 1). Firstly, 0.5 M PAANa/Na_2_SO_4_ gel electrolyte was prepared; 3.551 g of Na_2_SO_4_ and 0.8 g of PAANa were added into 50 mL of DI water under stirring at room temperature until the solution became clear and transparent. The nylon/Ag/MnO_2_ positive electrode and nylon/Ag/PPy negative electrode were dipped in the 0.5 M Na_2_SO_4_ aqueous solution, and then immersed into PAANa/Na_2_SO_4_ gel electrolyte for 2 min. Subsequently, two electrodes coated with gel electrolyte were placed on the flexible polyethylene terephthalate (PET) plate substrate in parallel with a spacing distance of 0.5 mm, while two pieces of conductive copper tape were adhered to both ends of the PET plate as the electrode tabs of the device. The gap between the two electrodes and the surfaces of the electrodes were filled and covered with gel electrolyte. At last, the electrodes were covered with a thin polyethylene (PE) protection film for device packaging. Eventually, the nylon/Ag/MnO_2_||nylon/Ag/PPy solid-state WASC was obtained with an effective length of 5 cm and area of 1.07 cm^2^.

### 2.5. Materials Characterizations

The surface morphologies of the fabricated electrodes were characterized by a scanning electron microscope (SEM, ZEISS Crossbeam 350, Jena, Germany) equipped with energy-dispersed X-ray spectroscopy (EDS). The surface roughness of nylon yarn and nylon/Ag yarn were measured by a ContourGT-X three-dimensional profilometer. A Nicolet IS10 FT-IR spectrometer was used to obtain the Fourier-transformed infrared spectra (FT-IR) of the fabricated electrodes. Additionally, X-ray photoelectron spectroscopy (XPS) measurements were performed using an Axis SUPRA + spectrometer with a monochromated Al Kα radiation (*hv* = 1486.6 eV).

### 2.6. Electrochemical Measurements

The electrochemical performance of the fabricated electrodes was carried out in an electrochemical workstation (CHI660E) produced by Chenhua (Shanghai, China) with a three-electrode configuration in 0.5 M Na_2_SO_4_ aqueous electrolyte. In this three-electrode configuration, the fabricated electrode, a platinum foil and a SCE were served as the working electrode, counter electrode and reference electrode, respectively. The electrochemical performance of the assembled WASC was tested via a two-electrode system. Cyclic voltammetry (CV) and galvanostatic charge–discharge (GCD) tests were conducted at various scan rates and currents. Electrochemical impedance spectroscopy (EIS) measurements were performed in the frequency range from 0.01 Hz to 100 kHz with a 5 mV amplitude.

The capacitance (C), C_L_ and C_A_ of the fabricated electrode and the assembled device were calculated from the data of the CV curve and the GCD curve based on the following equations:

(1) $$C=\frac{\int i\;\mathrm{dV}}{v\Delta U}$$

(2) $$C=\frac{I\Delta t}{\Delta V}$$

(3) $$C_{L}=\frac{\int i\;\mathrm{dV}}{v\Delta U\times L}$$

(4) $$C_{L}=\frac{I\Delta t}{\Delta V\times L}$$

(5) $$C_{A}=\frac{\int i\;\mathrm{dV}}{v\Delta U\times A}$$

(6) $$C_{A}=\frac{I\Delta t}{\Delta V\times A}$$

where i, v, ΔU and L represent the current (A), scan rate (V s^−1^), potential range (V) of CV test and effective length (cm) of the fabricated electrode or device, respectively. I, Δt, ΔV and A indicate the discharging current (A), discharge time (s), potential range (V) of the GCD test and the effective surface area of the fabricated electrode or device (cm^2^), respectively [25].

The areal energy density (E_A_) and the areal power density (P_A_) of the device were calculated from the following equations:

(7) $$E_{A}=\frac{C_{A,\mathrm{device}}\times {\Delta U}^{2}}{2\times 3600}$$

(8) $$P_{A}=\frac{3600E_{A}}{\Delta t}$$

C_A,device_ is the areal specific capacitance of the device calculated from Equation (6), and ΔU and Δt are the potential range (V) and discharge time (s) of the device in the GCD test, respectively.

## 3. Results

**Figure 2:**
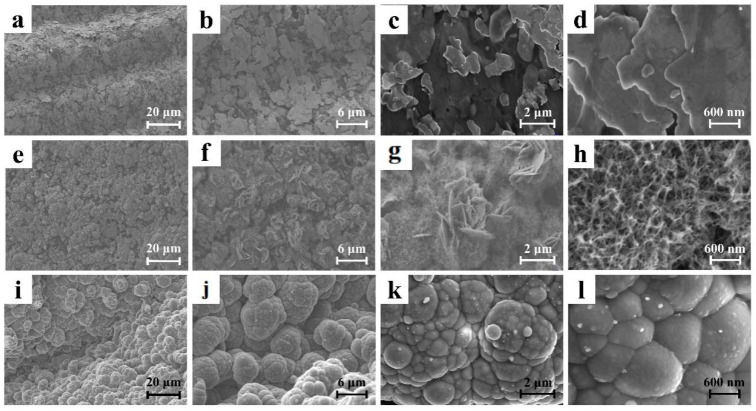
SEM images of (**a**–**d**) nylon/Ag; (**e**–**h**) nylon/Ag/MnO_2_ and (**i**–**l**) nylon/Ag/PPy electrodes at different magnifications.

The surface morphologies of the nylon yarn, the nylon/Ag current collector, nylon/Ag/MnO_2_ and the nylon/Ag/PPy electrode were characterized by SEM. As shown in Figure S3, multiple thin nylon fibers with an average diameter of ~16.3 µm were twisted into nylon yarn, which is beneficial for the mechanical performance. Figure 2a–d show that the surface of the nylon yarn was uniformly covered by layers of stacked silver nanoflakes, which is quite different from the original smooth surface of the nylon yarn shown in Figure S3. This silver coating layer eventually lowered the resistance of the nylon/Ag yarn. Figure 2e–h display the SEM images of the surface of the nylon/Ag/MnO_2_ electrode. The MnO_2_ 2-D nanosheets (with an average thickness of ~16 nm) and 3-D nanoflowers can be observed on the electrode surface as shown in Figure 2f–h. This type of loose and porous structure could favor the access of electrolyte and provide more active sites for ions adsorption [26]. The surface morphology images of as-fabricated nylon/Ag/PPy electrodes are shown in Figure 2i–l. After chemical polymerization and electrochemical polymerization, PPy was coated on the surface of nylon/Ag yarn with numerous cauliflower-shaped microstructures. This spherical cauliflower structure may expand the contact area between the electrode and electrolyte, which promotes the capacitance of nylon/Ag/PPy [27].

**Figure 3:**
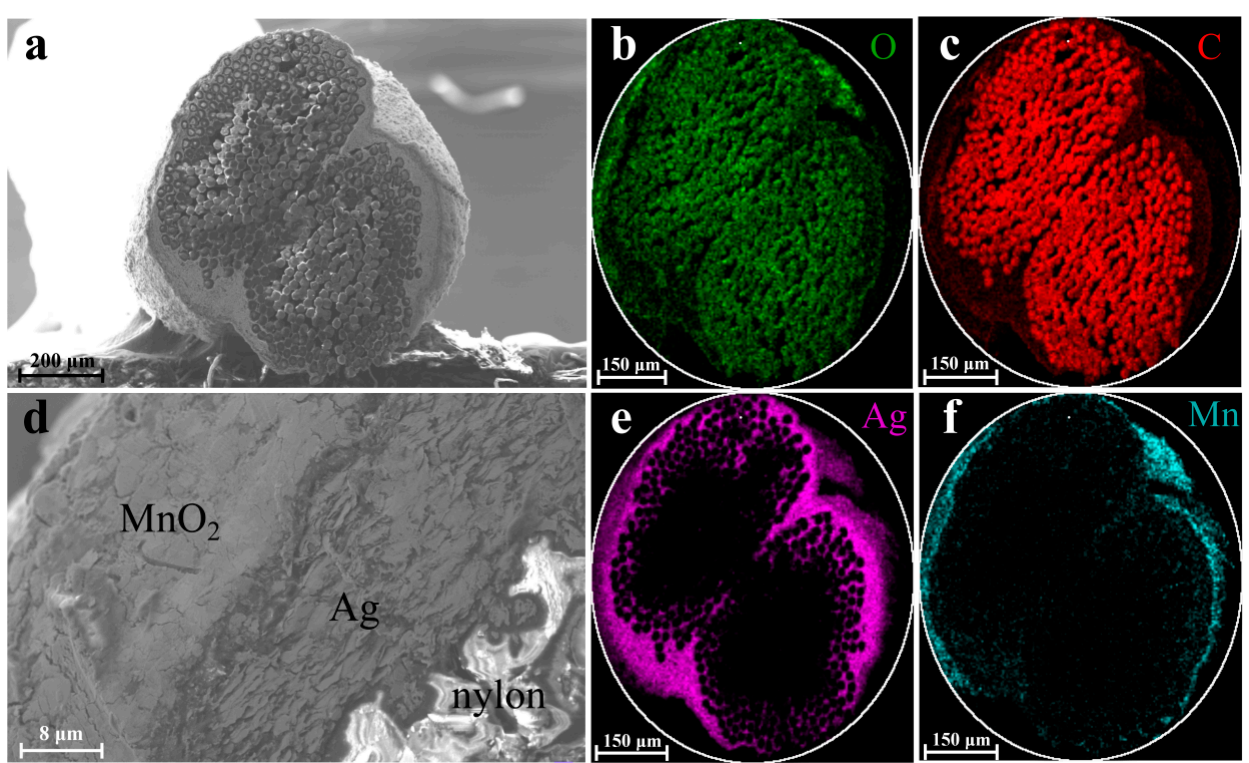
(**a**) Cross-sectional SEM images of the nylon/Ag/MnO_2_ electrode; (**b**) oxygen and (**c**) carbon element distribution in the cross-section derived from EDS mapping scan results of the nylon/Ag/MnO_2_ electrode; (**d**) different material layers of the cross-section of the nylon/Ag/MnO_2_ electrode; (**e**) silver and (**f**) manganese element distribution in the cross-section.

**Figure 4:**
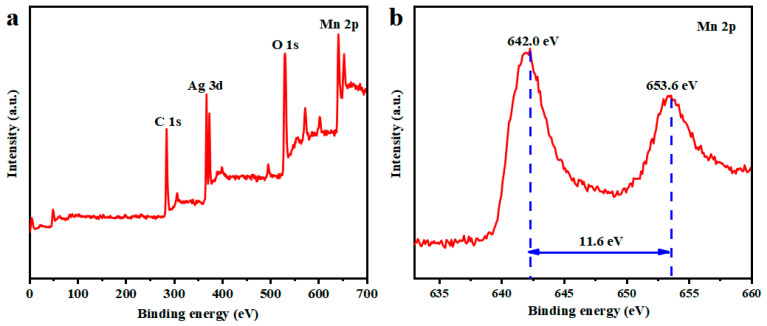
(**a**) Wide-scan XPS spectra of nylon/Ag/MnO_2_; (**b**) narrow spectra of Mn 2p peaks.

To figure out the composition of each yarn electrode, the cross-sectional morphologies of the nylon/Ag/MnO_2_ and nylon/Ag/PPy electrodes were characterized by SEM and the corresponding EDS mapping scan; the results are displayed in Figure 3 and Figure 4. Figure 3a shows the cross-sectional morphology of the nylon/Ag/MnO_2_ electrode, in which a coaxial structure was constructed. Figure 3d demonstrates the boundaries and morphological distinctions between different layers at a higher magnification, in which the non-conductive nylon fibers at the bottom right corner appeared blurred because of the charging effect caused by the electron irradiation [28]. Figure 3b,c show the distributions of O and C in the central area, which correspond to the nylon yarn. Figure 3e shows the Ag element at the surrounding area of the nylon yarn, which indicates that the silver paste was coated onto the nylon yarn substrate uniformly with a small amount of Ag paste penetrating into the superficial layer of the nylon yarn. Meanwhile, Mn and O elements existed in the outer area, most of which overlapped with Ag. During the process of electrochemical deposition, nylon/Ag served as the working electrode and the substrate for MnO_2_ to grow on, Mn^2+^ entered the interior of the silver layer and MnO_2_ was almost electrochemically deposited on the surface of Ag layer, eventually resulting in the intimate contact of Ag layer and MnO_2_ layer. Therefore, the different layers from inside to outside are nylon yarn, the Ag layer and the MnO_2_ layer, respectively (Figure 3d).

The XPS spectrum of the nylon/Ag/MnO_2_ electrode is shown in Figure 4. In Figure 4a, the peaks of Ag 3d, O 1s, C 1s and Mn 2p can be observed. Additionally, the peaks of Mn (2p_1/2_, 2p_3/2_) are displayed in Figure 4b. The Mn 2p_1/2_ and 2p_3/2_ peak values were located at 642.0 eV and 653.6 eV, respectively, with a separated spin energy of 11.6 eV. These results are in accordance with previous reported data of Mn 2p_1/2_ and 2p_3/2_ in MnO_2_ [29,30]. It can be confirmed that MnO_2_ was successfully electrodeposited on nylon/Ag.

**Figure 5:**
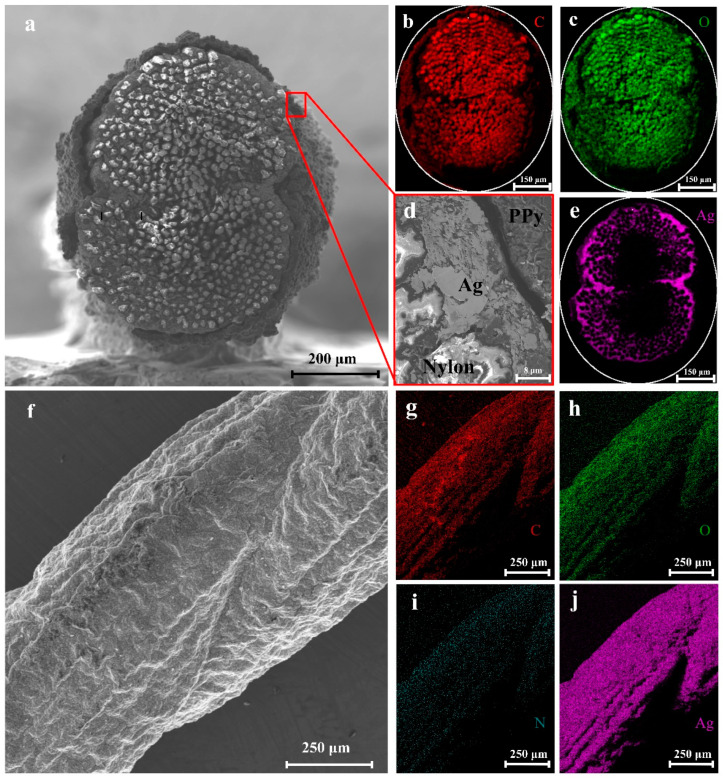
(**a**) Cross-sectional SEM image of the nylon/Ag/PPy electrode; (**b**) carbon and (**c**) oxygen element distribution in the cross-section; (**d**) different material layers of the cross-section of the nylon/Ag/PPy electrode; (**e**) silver element distribution in the cross-section; (**f**) lateral SEM image of the nylon/Ag/PPy electrode; lateral (**g**) carbon (**h**), oxygen (**i**), nitrogen and (**j**) silver element distribution.

The cross-sectional and surface morphology of the nylon/Ag/PPy electrode is displayed in Figure 5a,f, while Figure 5b,c,e show EDS mapping of the cross-section of the electrode. Figure 5g–j show the EDS mapping of the surface of the nylon/Ag/PPy electrode. As shown in Figure 5b,c,g,h, O and C elements mainly existed in the central area, corresponding to the nylon yarn, while C elements at the exterior of the cross-section of the nylon/Ag/PPy outer area and N and C elements at the surface of the electrode were the main components of PPy covered on the Ag layer. It can also be seen that the Ag pastes were coated on the nylon yarn and penetrated into the interior of the nylon yarn as shown in Figure 5e,j. It is seen that there is nylon yarn, an Ag layer and a PPy layer from the inside to outside (Figure 5a,d). Figure S2b shows that the PPy layer cannot be easily polymerized on the relatively smooth Ag layer (Ra = 2.8 μm, Figure S4a) through direct electrochemical polymerization. In fact, the rougher the substrate is, the more adsorption sites for monomers can be provided [31]. After chemical polymerization, the surface roughness of nylon/Ag/PPy was dramatically increased (Ra = 5.2 μm, Figure S4b); thus, the PPy could be easily coated on the chemically polymerized nylon/Ag/PPy by electrochemical polymerization, and the obtained PPy layers were more uniform than those of nylon/Ag/PPy prepared by direct electrochemical polymerization (Figure S2b). This novel process is considered as an effective way to prepare PPy on the smooth surface of substrates.

**Figure 6:**
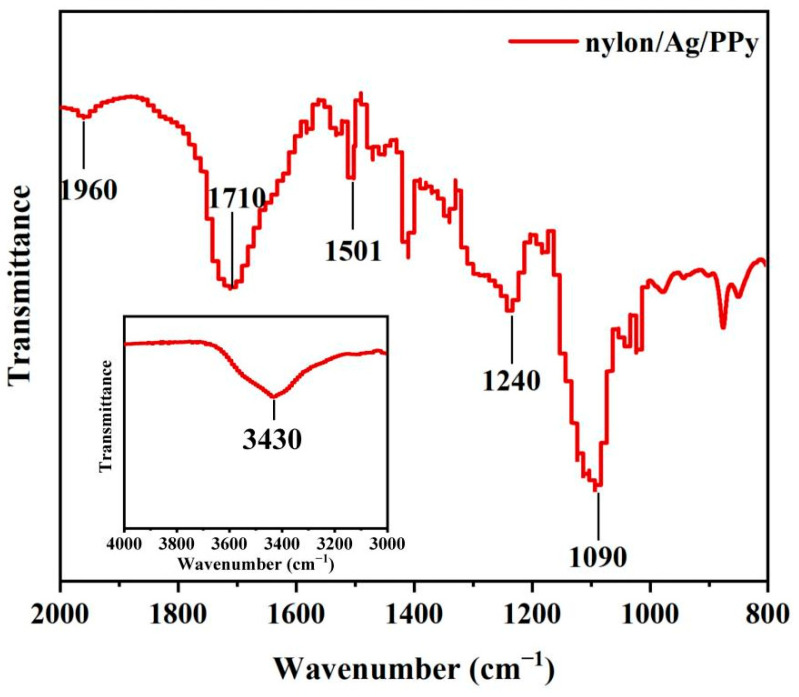
FT-IR spectra of the nylon/Ag/PPy electrode.

The FT-IR patterns of the nylon/Ag/PPy electrode were shown in Figure 6. The absorption peaks at 1090 cm^−1^ and 1240 cm^−1^ are attributed to C-N stretching vibrations of polypyrrole [32]. The characteristic peaks at 1501 and 1710 cm^−1^ correspond to the PPy ring stretching characteristic bands [33]. The peak at 1960 cm^−1^ comes from the N-H deformation vibration absorption peak on the polypyrrole ring [32]. Furthermore, the band at 3430 cm^−1^ describes C-H stretching vibrations [33]. These characteristic peaks confirmed that the PPy was successfully synthesized on the surface of the nylon/Ag yarn.

**Figure 7:**
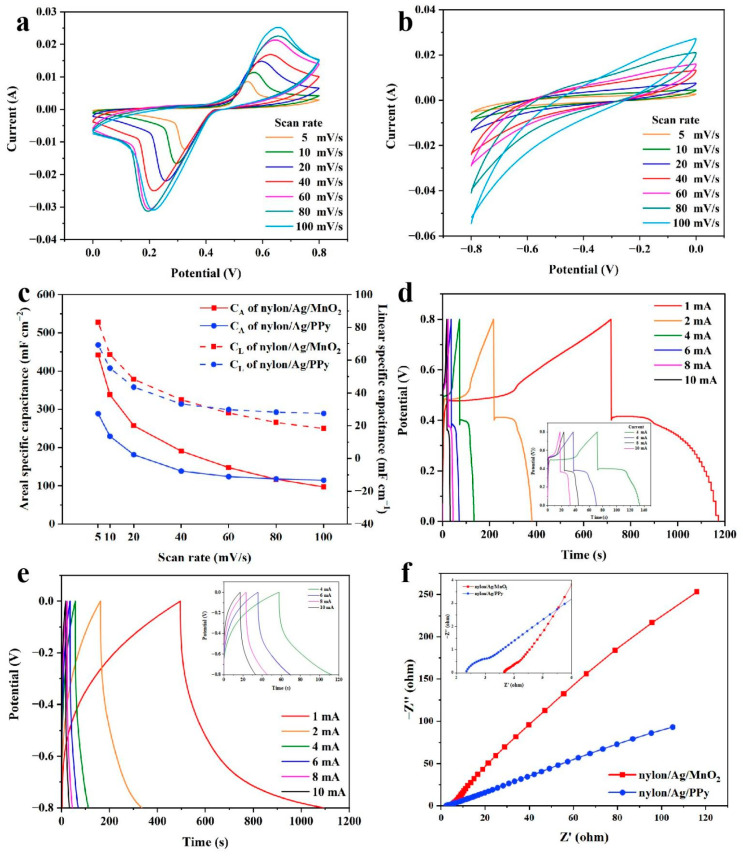
(**a**,**b**) CV curves of the (**a**) nylon/Ag/MnO_2_ and (**b**) nylon/Ag/PPy electrodes at different scan rates; (**c**) the C_L_ and C_A_ calculated from CV curves in (**a**,**b**) of the two electrodes at different scan rates; (**d**,**e**) GCD curves of (**d**) nylon/Ag/MnO_2_ and (**e**) nylon/Ag/PPy electrodes at different charge/discharge currents (the inset shows the GCD curves from 4 mA to 10 mA); (**f**) EIS of the electrodes (the inset shows the enlarged view of the high-frequency region).

The electrochemical performances of the prepared electrodes were evaluated by CV, GCD and EIS tests with a three-electrode configuration. Figure 7a,b show the CV curves of nylon/Ag/MnO_2_ and nylon/Ag/PPy at different scan rates ranged from 5 mV s^−1^ to 100 mV s^−1^ in the potential window of 0 V to 0.8 V and −0.8 V to 0 V, respectively. As shown in Figure 7a, obvious oxidation peaks and reduction peaks can be observed in the CV curves of nylon/Ag/MnO_2_; this can be ascribed to the surface Faradic reactions between the Na_2_SO_4_ electrolyte and the MnO_2_ active material, which finally results in the pseudocapacitive characteristic of MnO_2_. This process can be demonstrated by the following equation [34]:

(9) $${\mathrm{MnO}}_{2}+\delta X^{+}+\delta e^{-}\underset{\overset{\leftarrow }{\mathrm{Discharge}}}{\overset{\mathrm{Charge}}{\to }}{\mathrm{MnOOX}}_{\delta }$$

where X^+^ represents the H^+^ or alkaline metal cation, such as Na^+^, Li^+^ or K^+^ (Na^+^ in this work). Additionally, it can be seen in Figure 7a that the reduction/oxidation peaks shifted towards 0 V/0.8 V and became broader as the scan rate increased. The peak potential (E_p_) shifting can be attributed to the polarization of the electrode, which broadened the distance between the oxidation peak and the reduction peak [35]. The peak current (I_p_) shifting can be explained by the proportional relationship between I_p_ and the square root of the scan rate (v^1/2^) [36]. These two reasons resulted in the peak shifting when v varied from 5 mV s^−1^ to 100 mV s^−1^.

Figure 7b presents the CV curves of the nylon/Ag/PPy electrode at different scan rates in the potential range of −0.8 V to 0 V, exhibiting a different shape from the typical rectangular for double-layer capacitance, which implies the pseudocapacitive characteristic of PPy. Meanwhile, the current increased with the increasing of scan rate, maintaining the same shape at different scan rates, which indicates a stable behavior of the nylon/Ag/PPy electrode [37]. Based on their CV curves at different scan rates, the C_L_ and C_A_ of two electrodes were calculated and exhibited in Figure 7c. The C_L_ and C_A_ of both electrodes decreased as the scan rate increased. When the scan rate increased from 5 mV s^−1^ to 100 mV s^−1^, the C_A_ of nylon/Ag/MnO_2_ decreased from 442.5 mF cm^−2^ to 97.3 mF cm^−2^, and the corresponding C_L_ decreased from 83.2 mF cm^−1^ to 18.3 mF cm^−1^, while the C_A_ of nylon/Ag/PPy decreased from 288.7 mF cm^−2^ to 114.6 mF cm^−2^, and C_L_ decreased from 69.3 mF cm^−1^ to 27.5 mF cm^−1^. This decreasing trend could be explained by the inadequate ion transport time caused by the increase in scan rate, which led to a lower number of electroactive ions concentrated on the electrode/electrolyte surface and lower utilization of the surface of active materials [38]. It should be noted that six CV scans were performed in this work, and the third scan is depicted in Figure 7a,b.

Figure 7d exhibits the GCD curves of nylon/Ag/MnO_2_; the inset shows the GCD curves at 4 mA to 10 mA. The nonlinear shape of the curves indicates that the capacitance of nylon/Ag/MnO_2_ electrode mainly comes from the Faradic reaction, and this is in accordance with its CV curves. A relatively large IR_drop_ in GCD curves of nylon/Ag/MnO_2_ can be observed. ESR can be given by ΔIR_drop_/Δi, which is determined by the slope of the fitted linear curve of IR_drop_ versus the discharge current. Figure S5 shows a low slope of the fitted curve of IR_drop_ versus the discharge current, which indicates the low ESR of nylon/Ag/MnO_2_ electrode (about 4.4 Ω) [39,40]. The GCD curves of the nylon/Ag/PPy electrode are shown in Figure 7e. The linear charge/discharge curves reveal the good capacitive characteristic of the nylon/Ag/PPy electrode. Figure 7f shows the Nyquist plots of two fabricated electrodes; the inset shows the enlarged view of the high-frequency region. The intercept between the semicircle and the real axis reflects the equivalent series resistance (ESR) of the electrodes, which includes the ionic resistance of the electrolyte, the intrinsic resistance of the active material and the contact resistance of the electrode/collector interface [41]. The ESR value of nylon/Ag/MnO_2_ was about 3.6 Ω, which is close to the ESR value obtained from the slop of fitted linear curve of IR_drop_ versus the discharge current in Figure S5. The ESR of the nylon/Ag/PPy was about 2.4 Ω. The diameter values of the semicircles correspond to the ionic or charge transfer resistances (R_ct_) of the electrodes, which were 1.0 Ω for nylon/Ag/MnO_2_ and 1.1 Ω for nylon/Ag/PPy. The lower-frequency region of nylon/Ag/MnO_2_ and nylon/Ag/PPy both showed a straight line, which can be ascribed to the processes mainly controlled by ion diffusion [42].

**Figure 8:**
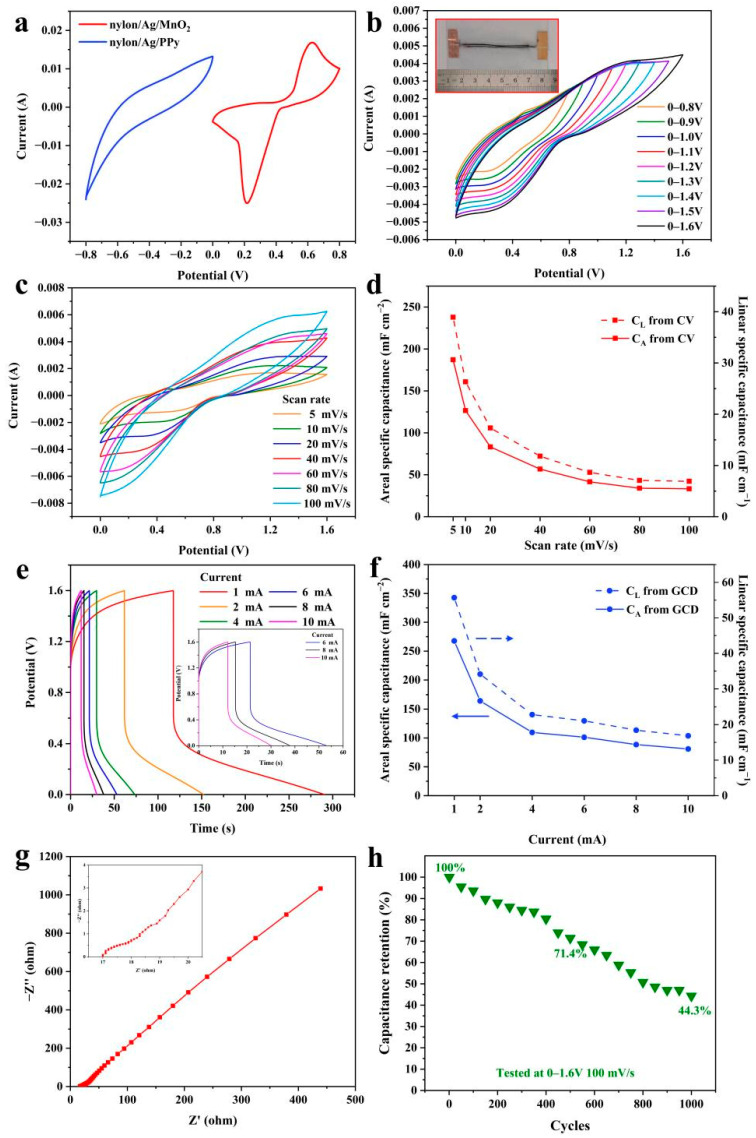
(**a**) Comparison of CV curves of the two electrodes at the scan rate of 40 mV s^−1^; (**b**) CV curves of the device at 40 mV s^−1^ in various potential windows (the inset shows the optical picture of the fabricated device); (**c**) CV curves of the device at different scan rates; (**d**) the C_L_ and C_A_ of the device calculated from CV curves; (**e**) GCD curves of the device at different charge/discharge currents (the inset shows the GCD curves from 6 mA to 10 mA); (**f**) the C_L_ and C_A_ of the device calculated from GCD curves; (**g**) EIS (the inset shows the enlarged view of the high-frequency region); (**h**) cycling stability test of the device at a scan rate of 100 mV s^−1^.

The CV curves of the two fabricated electrodes at the scan rate of 40 mV s^−1^ were compared and the results are shown in Figure 8a. The areas enclosed by the two CV curves were approximately equal, which means the capacitances were very close (179.5 mF for nylon/Ag/MnO_2_, and 166.5 mF for nylon/Ag/PPy; the corresponding C_L_ are 35.9 mF cm^−1^ and 33.3 mF cm^−1^). To fabricate a solid-state wire-shaped asymmetric supercapacitor, the prepared nylon/Ag/MnO_2_, nylon/Ag/PPy and PAANa/Na_2_SO_4_ gel electrolyte were used as the positive electrode, negative electrode and electrolyte, respectively. To maximize the performance of the WASC, it is necessary to achieve charge balance between the two electrodes. The charge storage (*Q*) of the two electrodes can be derived from Equation (10) [43]:

(10) $$Q=C_{L}L\Delta V$$

where *Q* is the charge stored in the electrodes, C_L_ is the linear specific capacitance of the electrodes, L is the length of the electrodes adopted in the WASC and ΔV is the potential range of each electrode. To reach *Q*^+^ = *Q*^−^, the length ratio of the two electrodes was expressed as the following equation:

(11) $$\frac{L^{+}}{L^{-}}=\frac{C_{L}^{-}\Delta V^{-}}{C_{L}^{+}\Delta V^{+}}$$

The parameters with the superscripts “+” and “−”correspond to the positive and negative electrodes. Based on the above-shown equation, the C_L_ of nylon/Ag/MnO_2_ and nylon/Ag/PPy based on the CV curves in Figure 8a was used; the length ratio of the negative and positive electrodes was close to 1:1. Therefore, both a nylon/Ag/MnO_2_ electrode and a nylon/Ag/PPy electrode with lengths of 5 cm were used to assemble the WASC, whose optical picture is shown in the inset of Figure 8b. The electrochemical performances of the constructed WASC were studied and the results are shown in Figure 8b–h. Figure 8b displays the CV curves of the assembled WASC at 40 mV s^−1^, while the potential window varied from 0–0.8 V to 0–1.6 V. The CV curves retained a similar shape and no obvious deformation was observed even when the potential reached 1.6 V, implying the good capacitive behavior of the WASC in this broad potential window. With the voltage windows extended from 0.8 to 1.6 V, the calculated C_A_ of the device increased from 37.5 mF cm^−2^ to 58.0 mF cm^−2^, and the corresponding energy density of the WASC increased about six-fold from 3.4 μWh cm^−2^ to 21.1 μWh cm^−2^.

Figure 8c shows the CV curves of the assembled WASC at different scan rates from 5 mV s^−1^ to 100 mV s^−1^ between 0 V and 1.6 V. The weak and broad redox peaks can be ascribed to the pseudocapacitive characteristic of the supercapacitor generated by the Faradic reaction of MnO_2_ and PPy [14]. The C_A_ and C_L_ calculated from the CV curves (Figure 8c) are displayed in Figure 8d. The value of C_A_ can reach 181.7 mF cm^−2^ (C_L_, 38.9 mF cm^−1^) at the scan rate of 5 mV s^−1^. The rough surface and porous structure of both electrodes allow the rapid insertion and extraction of electrolyte ions [44], which help to generate high capacitance of the fabricated supercapacitor. When the scan rate increased to 100 mV s^−1^, the C_A_ and C_L_ decreased to 32.3 mF cm^−2^ and 6.9 mF cm^−1^ because the ions in the working electrolyte cannot reach the inner surface of the active materials with enough time at a high scan rate. The GCD curves of the WASC at different currents (1 mA to 10 mA) are shown in Figure 8e, while the inset shows the GCD curves at 6 mA to 10 mA. Based on the GCD curves, the C_L_ and C_A_ were calculated and the results are shown in Figure 8f, delivering a high C_A_ of 260.0 mF cm^−2^ (C_L_ of 55.7 mF cm^−1^) at the current of 1 mA, and retaining at 78.7 mF cm^−2^ (C_L_ of 16.7 mF cm^−1^) when the current rose to 10 mA.

To further investigate the electrochemical properties of the WASC, the EIS test was carried out and is shown in Figure 8g. The low values of ESR (17.0 Ω) and R_ct_ (1.5 Ω) can be attributed to the low linear resistance of nylon/Ag yarn and the good electrolyte permeation of the electrodes [44]. Furthermore, the cyclic stability of the WASC was conducted through CV test at the scan rate of 100 mV s^−1^ between 0 V–1.6 V and the results are displayed in Figure 8h. During the 1000 cycles, the data of the CV test were recorded every 50 cycles. As the cycle number increased, the capacitance retention decayed, remaining at 71.4% after 500 cycles and 44.3% after 1000 cycles. The lower retention of the WASC can be attributed to the following reasons. Firstly, the mechanism of expansion and contraction of the polymer chain in PPy during the ion doping/de-doping process when charging and discharging decreases the conductivity and capacitance of the WASC [45]. Secondly, MnO_2_ materials store charges via the redox reaction explained in Equation (9); by changing the phase of Mn, charges can be transported between the electrode and electrolyte. The incompletely irreversible redox reaction induces the inherent instability of MnO_2_ material, resulting in the obvious decrease in cycle stability [46].

**Figure 9:**
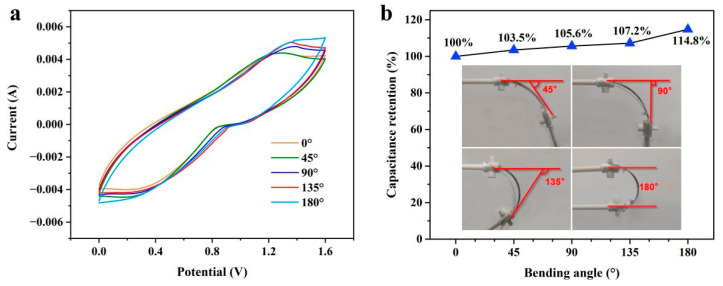
(**a**) CV curves of the WASC device under different bending angles at the scan rate of 40 mV s^−1^; (**b**) the capacitance of the device at different bending angles.

To evaluate the electrochemical stability of the WASC under bending state, the CV characteristic was tested at 40 mV s^−1^ under different bending angles from 0° to 180°, which is shown in Figure 9a. Two platinum clips were used to adjust the bending angle of the WASC as shown in the inset of Figure 9b. During the CV tests, two clips were connected to the WASC and the positive and negative electrodes of the electrochemical workstation, respectively. It can be seen that the CV curves showed approximately the same shape at different bending angles and the capacitance of the tested WASC varied within 4% as the bending angle increased (Figure 9b), demonstrating the good electrochemical stability of the WASC under the bending state.

**Figure 10:**
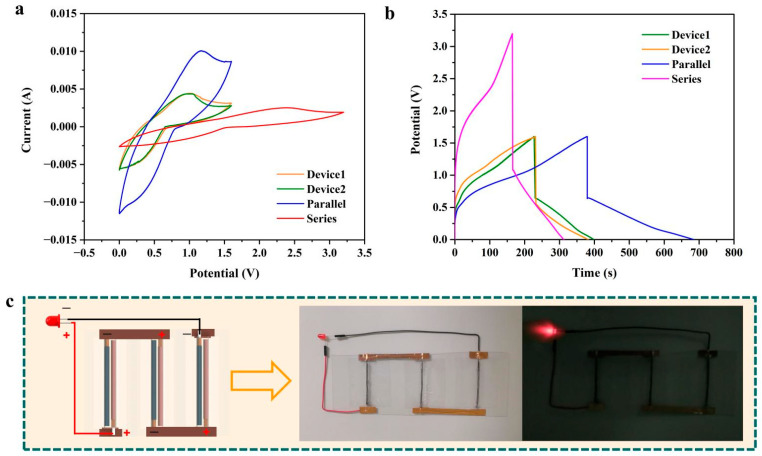
(**a**) CV curves. (**b**) GCD curves of two fabricated devices in parallel and series connections; (**c**) schematic diagram and optical picture of the circuit consisting of three as-fabricated WASCs to illuminate a LED.

Considering the practical application of fabricated WASCs, it is essential to increase their operating voltage and current by connecting them in parallel and in series. Thus, the electrochemical performances of two as-fabricated supercapacitors (denoted as Device 1 and Device 2) connected in parallel (denoted as Parallel) and in series (denoted as Series) were tested and analyzed. Figure 10a shows the CV curves of Device 1, Device 2, Parallel and Series at the scan rate of 40 mV s^−1^. The CV curves of Device 1 and Device 2 were very close, and their capacitances were 61.4 mF and 60.8 mF, respectively. Table 1 shows the comparison of the theoretical values and experimental values of Device 1, Device 2 and their combinations. It is worth noting that the capacitance measured in series is 32.4 mF, which is close to the theoretical value of 30.5 mF of two devices connected in series. When connecting two supercapacitors in parallel, the capacitance value reached 137.7 mF, which is also close to the theoretical value (122.2 mF). Figure 10b shows the GCD curves of Device 1, Device 2, Parallel and Series at the current of 1 mA. The GCD curves of Device 1 and Device 2 showed similar shapes, and the experimental capacitance values for Device 1 and Device 2 were close (260.9 mF and 272.6 mF, respectively). In addition, the calculated capacitance values for Series and Parallel based on the GCD Curve are consistent with the theoretical values (Table 1). The slight difference between Device 1 and Device 2 indicated the good repeatability of fabrication of the WASCs in this work. The small difference between the experimental values and theoretical values of capacitance revealed the excellent stability of the WASCs in series or parallel connection, meanwhile demonstrating their great potential in power wearable/portable electronics. Figure 10c shows the diagrammatic sketch and optical pictures of a simple application of the WASCs fabricated in this work. In this circuit, three as-fabricated WASCs were directly placed on a PET plate and connected in series with copper wires and copper tapes. It can be seen that a LED was successfully illuminated by these three charged WASCs in series, proving the applicability of the WASCs fabricated in this work.

**Figure 11:**
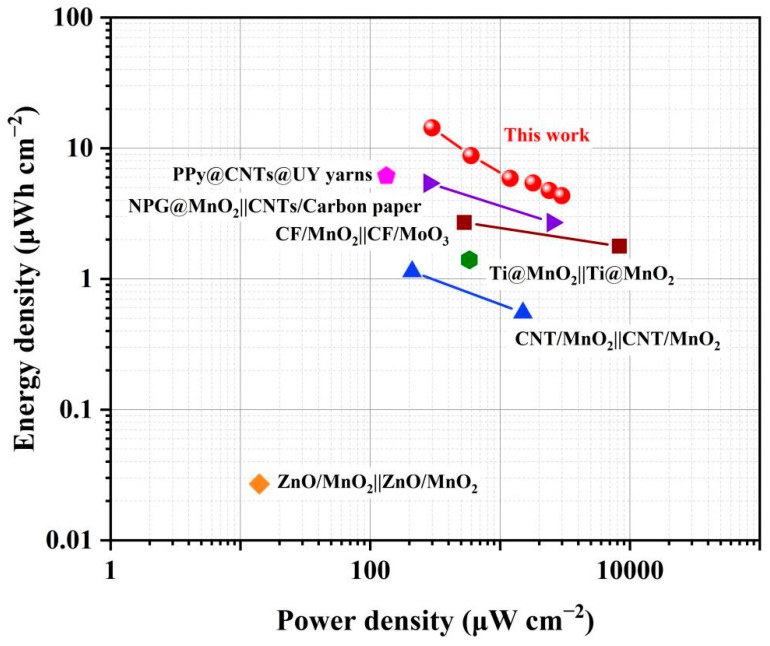
Ragone plot of the WASC compared with similar reported works.

Furthermore, Table 2 provides a brief review of the electrodes, configuration, gel electrolytes, potential windows and specific capacitance (C_L_ and C_A_) of some similar reported works, which also used MnO_2_ or PPy as active materials. It can be seen that although the voltage window of WASC in this work is not the highest among them, its C_L_ and C_A_ are higher than those of the flexible supercapacitors in other works. Compared with some identical symmetric pseudocapacitive devices such as CNT/MnO_2_||CNT/MnO_2_ [47], the C_L_ and C_A_ of the WASC in this work are clearly higher, and even when compared with asymmetric pseudocapacitive devices (CF/MnO_2_||CF/MoO_3_ [43], Cu wire/CuO NWs||CF/PPy [48] and CF/PPy||CNT/MnO_2_ [49], it still has better capacitive performance, which can be attributed to its excellent pseudocapacitive performance. Figure 11 presents the Ragone plot of the nylon/Ag/MnO_2_||nylon/Ag/PPy solid-state asymmetric supercapacitor calculated from GCD curves. With the increase in charge/discharge current, the areal energy density of the device varied from 13.9 to 4.2 μWh cm^−2^, corresponding to the power densities of 290 to 2902 μW cm^−2^. This performance is better than some previously reported works, such as the fiber/wire shaped supercapacitor of PPy@CNTs@UY yarns (6.13 μWh cm^−2^ at 133 μW cm^−2^) [50], NPG@MnO_2_||CNTs/Carbon paper (5.4–2.7 μWh cm^−2^ at 284–2531 μW cm^−2^) [51], CF/MnO_2_||CF/MoO_3_ (2.7–1.78 μWh cm^−2^ at 530–8300 μW cm^−2^) [43], Ti@MnO_2_||Ti@MnO_2_ (1.4 μWh cm^−2^ at 580 μW cm^−2^) [52], CNT/MnO_2_||CNT (1.14–0.55 μWh cm^−2^ at 210–1500 μW cm^−2^) [14] and ZnO/MnO_2_||ZnO/MnO_2_ (0.027 μWh cm^−2^ at 14 μW cm^−2^) [53]. Moreover, the performance of the WASC fabricated is also better than some supercapacitors which are based on compositing conducting polymers with metal nanoparticles and 2D materials, such as the micro-supercapacitor (MSC) based on Cu-OHTP/exfoliated graphene (EG) thin film (14.3 mF cm^−2^) [54] and MSC based on SA-MXene (108.1 mF cm^−2^) [55]. This excellent performance can be attributed to the high C_A_ and extended operating voltage window of the asymmetric supercapacitor prepared in this work.

## 4. Conclusions

In conclusion, a nylon/Ag/MnO_2_ yarn electrode was fabricated by electrochemically depositing MnO_2_ on a nylon/Ag yarn. The nylon/Ag/PPy yarn electrode was prepared by two steps of chemical polymerization first and followed by electrochemical polymerization. Subsequently, the nylon/Ag/MnO_2_ electrode (positive electrode), nylon/Ag/PPy electrode (negative electrode) and PAANa/Na_2_SO_4_ gel electrolyte were employed to successfully construct a novel WASC with a wider potential window of 1.6 V. Meanwhile, because of the active pseudocapacitive characteristics of two electrodes, the WASC exhibited high C_L_ (38.9 mF cm^−1^) and C_A_ (181.7 mF cm^−2^) at 5 mV s^−1^ from CV curves. Moreover, the WASC showed a high energy density varying from 13.9 to 4.2 μWh cm^−2^ with the corresponding power density changing from 290 to 2902 μW cm^−2^, which is superior to some reported wire-shaped symmetric/asymmetric supercapacitors. In addition, the device showed good electrochemical stability under different bending conditions, indicating the potential application of WASC in flexible wearable/portable devices.

## Figures and Tables

**Table 1:** Comparison of the theoretical values and experimental values of Device1, Device 2 and their combinations.

	CV Test (40 mV s^−1^)			GCD Test (1 mA)		
	Theoretical Value/mF	Experimental Value/mF	Error	Theoretical Value/mF	Experimental Value/mF	Error
Device1	-	61.4	-	-	260.9	-
Device2	-	60.8	-	-	272.6	-
Parallel	122.2	137.7	12.7%	533.5	499.5	−6.8%
Series	30.5	32.4	6.2%	133.3	136.5	2.4%

**Table 2:** Comparison of the electrochemical performance of this work and similar reported works.

Electrodes	Configuration	Gel Electrolyte	Potential Window (V)	C_L_ (mF cm^−1^)	C_A_ (mF cm^−2^)	Ref.
nylon/Ag/MnO_2_ ||nylon/Ag/PPy	Parallel	0.5 M PAANa/Na_2_SO_4_	0–1.6	38.9	181.7	This work
CNT/MnO_2_ ||CNT/MnO_2_	Parallel	2 M PVA/H_3_PO_4_	0–1.0	0.75	-	[47]
CF/MnO_2_||CF/MoO_3_	Twisted	1 M PVA/KOH	0–2.0	-	4.86	[43]
Cu wire/CuO NWs ||CF/PPy	Twisted	2 M PVA/KOH	0–1.3	24.91	39.67	[48]
CF/PPy ||CNT/MnO_2_	Coaxial	5 M PVA/LiCl	0–1.6	13.5	66.3	[49]

## Data Availability

The data that support the findings of this study are available from the corresponding author [Ruirong Zhang], upon reasonable request.

## Supplementary Materials

The following supporting information can be downloaded at: https://www.mdpi.com/article/10.3390/polym15071627/s1. Figure S1. The stress-strain curves of nylon samples; Figure S2. (a) Optical picture of the comparison of the nylon/Ag substrate before and after heat curing; (b) Optical picture of the electrode fabricated via directly electrochemically polymerizing PPy on nylon/Ag; Figure S3. SEM images of the nylon yarn: (a) 40× magnification; (b) 3000× magnification; Figure S4. 3D profiles of (a) the nylon/Ag yarn and (b) the nylon/Ag/PPy yarn after chemical polymerization; Figure S5. Variation of IR drop with different discharge currents of nylon/Ag/MnO_2_ electrode.
