# Supplementary material for: A Solid-State Wire-Shaped Supercapacitor Based on Nylon/Ag/Polypyrrole and Nylon/Ag/MnO2 Electrodes
Source: Polymers (Basel). 2023 Mar 24;15(7):1627. doi: 10.3390/polym15071627 (PMC10097388; doi:10.3390/polym15071627)
Supplement: Supplementary file 1 [file polymers-15-01627-s001.zip › polymers-2266912-supplementary.pdf]

# A solid-state wire-shaped supercapacitor based on nylon/Ag/polypyrrole and nylon/Ag/$MnO_2$ electrodes

Ruirong Zhang [1,*], Xiangao Wang [1], Sheng Cai [1], Kai Tao [1] and Yanmeng Xu [2]

1 Ministry of Education Key Laboratory of Micro/Nano Systems for Aerospace, School of Mechanical Engineering, Northwestern Polytechnical University, Xi'an 710072, China

2 Cleaner Electronics Group, College of Engineering, Design and Physical Sciences, Brunel University London, Uxbridge UB8 3PH, UK

*E-mail: ruirongzhang@nwpu.edu.cn

## Supplementary Figures

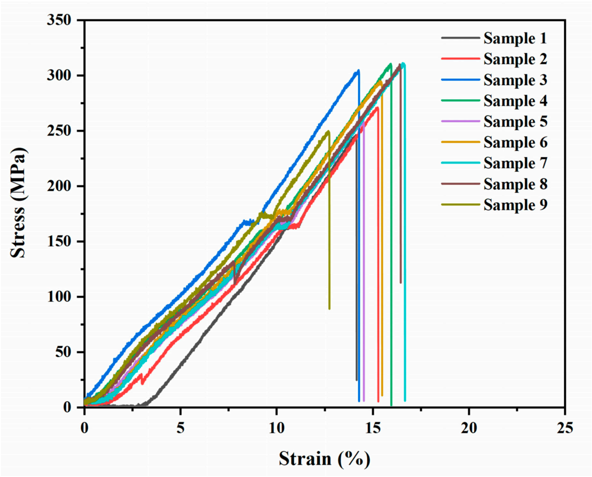


**Figure S1.** The stress-strain curves of nylon samples

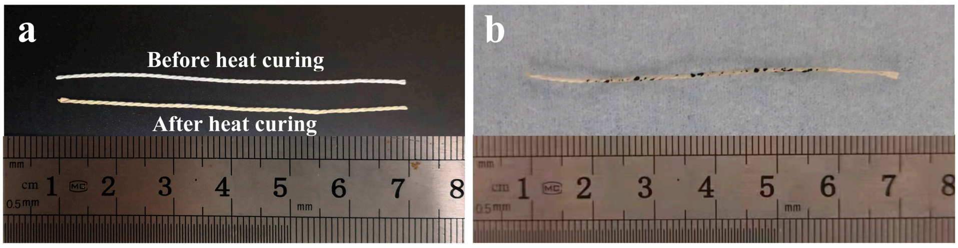


**Figure S2.** (a) Optical picture of the comparison of the nylon/Ag substrate before and after heat curing; (b) Optical picture of the electrode fabricated via directly electrochemically polymerizing PPy on nylon/Ag.

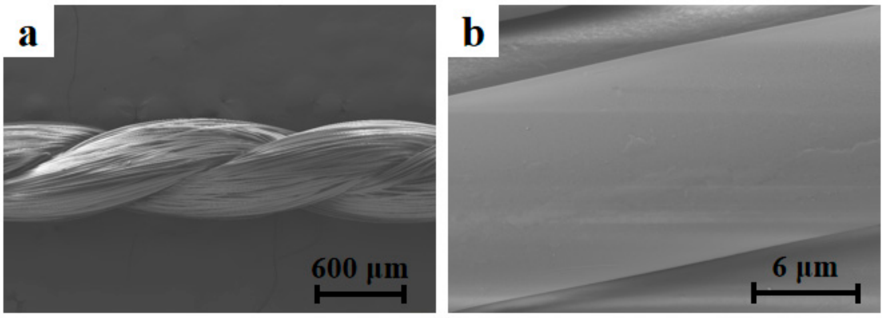


**Figure S3.** SEM images of the nylon yarn: (a) 40×magnification; (b) 3000×magnification.

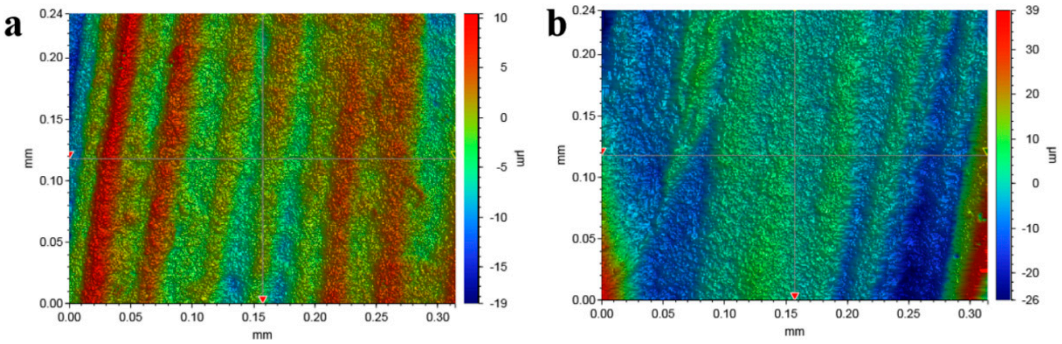


**Figure S4.** 3D profiles of (a) the nylon/Ag yarn and (b) the nylon/Ag/PPy yarn after chemical polymerization.

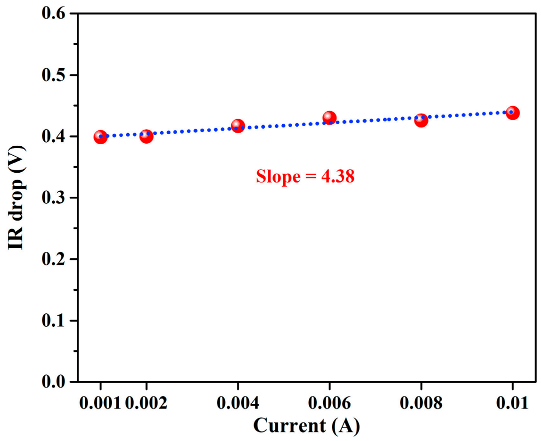


**Figure S5.** Variation of IR drop with different discharge currents of nylon/Ag/$MnO_2$ electrode.
